# Significance of Microglial Energy Metabolism in Maintaining Brain Homeostasis

**DOI:** 10.1007/s12975-022-01069-6

**Published:** 2022-07-26

**Authors:** John P. Bielanin, Dandan Sun

**Affiliations:** 1 Department of Neurology, University of Pittsburgh, Pittsburgh, PA 15213 USA; 2 Pittsburgh Institute for Neurodegenerative Disorders, University of Pittsburgh, Pittsburgh, PA 15213 USA; 3 Veterans Affairs Pittsburgh Health Care System, Pittsburgh, PA 15213 USA; 4 Department of Neurology, University of Pittsburgh Medical School, 7016 Biomedical Science Tower-3, 3501 Fifth Ave., Pittsburgh, PA 15260 USA

## Microglial Physiological Functions

Microglia are the most prominent immune cells of the CNS and play a critical role in maintaining normal brain homeostasis through constant surveillance of the brain’s microenvironment [1–3]. Under physiological conditions, microglia act as professional phagocytes of the CNS and aid in shaping neural circuity and plasticity throughout development [1, 2]. Pattern recognition receptors (PPRs) expressed by microglia allow for recognition of tissue injury or disease through recognition of danger-associated molecular patterns (DAMPS) or pathogen-associated molecular patterns (PAMPS), which provide signals that regulate the change in microglial function from homeostatic to active [2]. However, in an injured brain, microglia are rapidly activated and undergo phenotypic changes that contribute to both cell damage and restoration [1, 2]. Classically activated microglia are involved in inflammatory responses following injury [1–3]. Proinflammatory microglia release inflammatory cytokines and factors, such as TNF-α, IL-1β, IL-6, and reactive oxygen species (ROS), which contribute to neuronal injury [1]. Conversely, alternatively activated microglia are beneficial for brain repair and clearing toxic debris from the microenvironment through the release of anti-inflammatory cytokines/chemokines, such as BDNF, VEGF-A, IL-8, and Arg1, that promote remyelination, angiogenesis, axon regeneration, and blood–brain barrier integrity [1]. Modulation of the phenotypical polarization states of microglia may allow for new therapeutic interventions for treating brain injuries and diseases.

## Microglial Immunometabolism

**Fig. 1:**
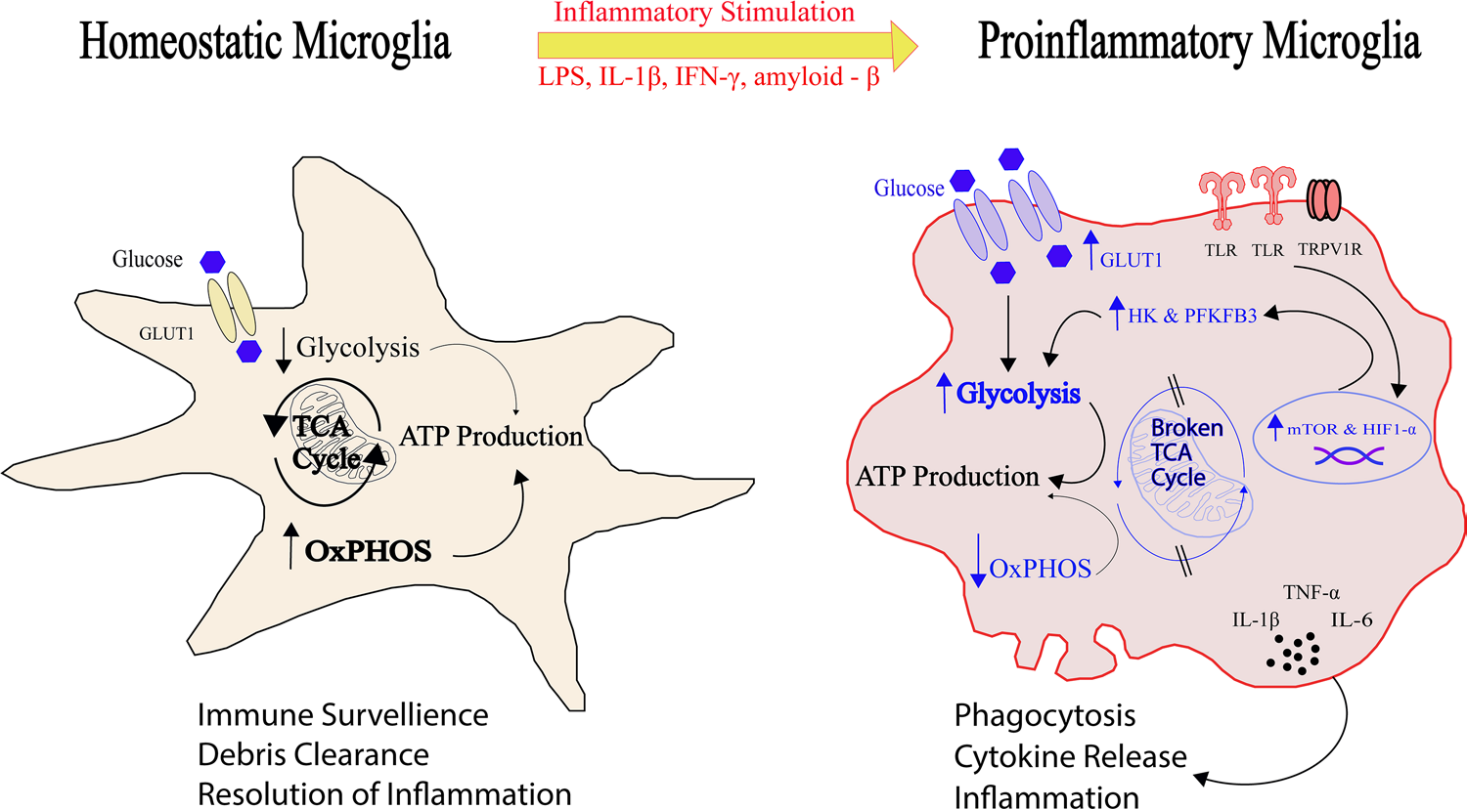
The metabolic homeostasis between OxPHOS and glycolysis is important for microglial function. Under physiological conditions, microglia primarily rely on OxPHOS for ATP production. In response to inflammatory stimulation, microglia undergo metabolic reprogramming to switch from OxPHOS to glycolysis. Metabolic reprograming was characterized by an increase in glycolysis, tricarboxylic acid (TCA) metabolite accumulation, hypoxia-inducible factor-1 α (HIF-1α), and mammalian target of rapamycin (mTOR) transcriptional control, and by a decrease in mitochondrial respiration, which subsequently regulates phagocytosis and cytokine production, etc. This figure was created with Adobe Illustrator software (Adobe Inc., Mountain View, CA USA). Abbreviations: *OxPHOS*, oxidative phosphorylation; *TCA*, tricarboxylic acid; *LPS*, lipopolysaccharide; *IL-1β*, interleukin-1β; *IFN-γ*, interferon-γ; *TLR*, toll-like receptor; *TRPV1R*, transient receptor potential vanilloid type 1 receptor; *mTOR*, mammalian target of rapamycin; *HIF1-1α*, hypoxia inducible factor-1α; *GLUT 1*, glucose transporter 1; *HK*, hexokinase; *PFKFB3*, 6-phosphofructo-2-kinase/fructose-2,6-bisphosphatase 3; *TNF-α*, tumor necrosis factor-α

Coordinated regulation of microglial activation and energy metabolism plays a central role in microglial function [3, 4]. Under resting conditions, microglia primarily rely on OxPHOS for ATP production [3–5] (Fig. 1). However, increasing evidence demonstrates that when exposed to proinflammatory stimuli (LPS, IL-1β, IFN-γ), microglia undergo metabolic re-programming to switch from OxPHOS to aerobic glycolysis (Fig. 1) [3–5]. LPS-activated BV-2 microglial cells increased lactate production and decreased mitochondrial O_2_ consumption and ATP production, indicating a higher reliance on aerobic glycolysis for energy use [4, 5]. The metabolic re-programming was characterized by an increase in glycolysis, tricarboxylic acid (TCA) metabolite accumulation, hypoxia-inducible factor-1 α (HIF-1α), and mammalian target of rapamycin (mTOR) transcriptional control, and by a decrease in mitochondrial respiration [4] (Fig. 1). The switch to glycolysis during microglial activation is suggested to involve changes in several key proteins, such as upregulation of glucose transporter protein GLUT 1, increased transcription of hexokinases (HK 1–3), and upregulation of 6-phosphofructo-2-kinase/fructose-2,6-bisphosphatase 3 (PFKFB3) [4]. Additionally, the mTOR-HIF-1α axis was elevated upon LPS treatment, suggesting its involvement in regulating metabolic control [4]. Pharmacological inhibition of glycolysis in BV-2 microglia with aerobic glycolytic inhibitors (2-DG and 3-bromopyruvic acid (3-BPA)) reduced LPS-induced proinflammatory gene expression [3–5]. These findings clearly demonstrate the close link between microglial glucose metabolism and proinflammatory activation.

Metabolic reprogramming during inflammation also induces morphological changes in mitochondria, such as mitochondrial fission [6]. Mitochondrial fission removes damaged mitochondria from cells and allows them to adapt to an increase in glycolytic demand [6]. The mechanism remains unknown; however, it may involve in accumulation of TCA intermediate succinate, which enhances reverse electron transport activity and allows for production of ROS [6]. Fatty acid (FA) and lipid metabolism may also contribute to metabolic re-programming in microglia [7]. Classically activated macrophages increase levels of FA synthesis, while alternatively activated macrophages increase levels of FA oxidation and OxPHOS [7]. Future studies of FA and lipid metabolism in microglia are needed to improve our understanding of their role in immunometabolism during neurological diseases.

## Changes of Microglial Metabolism in Brain Diseases

New studies have begun to investigate the relationship between microglial energy metabolism and function [6–10]. Following stroke, activated microglia release a variety of pro- and anti-inflammatory cytokines [6]. The binding of DAMPS to Toll-like receptors (TLRs) and P2XY receptors resulted in a shift from OxPHOS to glycolysis in responding microglial cells [6]. Increased concentrations of glycolytic intermediates, such as glucose-6-phosphate (G6P), fructose-6-phosphate (F6P), lactate, and pyruvate were all found in brain tissue following ischemic reperfusion injuries, suggesting a greater reliance on glycolysis in stroke brains [6]. Knockdown of the rate-limiting enzyme in glycolysis, HK2, in Sprague–Dawley rats demonstrated neuroprotective effects by suppressing activation of microglia and IL-1β production [6]. Furthermore, the suppression of glucose utilization through application of 2-DG, ketogenic diet therapy, and caloric restriction alleviated inflammation and reduced stroke infarct area [6, 10]. In a recent study with transgenic deletion of microglial pH-regulating protein NHE1 (Na^+^/H^+^ exchanger isoform 1) in Cx3cr1-Cre^ER+/−^, Nhe1^flox/flox^ mice, we detected a significantly enhanced microglial OxPHOS metabolism in stroke brains [8]. These changes in microglia led to improved synaptic modeling, myelin repair, and elevated phagocytic activity [8]. Interestingly, in response to traumatic brain injury, elevated levels of arachidonic acid led to the recruitment and activation of microglia, which were characterized by an increase in tryptophan metabolism and oxidative stress, further contributing to neuronal death and toxicity [7].

Activated microglia are also a major pathological feature of Alzheimer’s disease (AD) [9]. In early AD, microglia aid in amyloid- β (Aβ) plaque clearance; however, activated microglia in late AD paradoxically contribute to disease progression by decreasing responses to Aβ deposition [9]. Metabolic profiling of microglia demonstrated that Aβ acutely triggers microglial activation and metabolic reprogramming, shifting their metabolism from OxPHOS to aerobic glycolysis by increased flux through the mTOR-AKT-HIF-1α pathway [9]. Microglia subsequently reach a “chronic tolerant” phenotype, characterized by significantly suppressed energy metabolism and immune function via the mTOR-AKT-HIF-1α pathway [9]. Pharmacological activation of transient receptor potential vanilloid type 1 (TRPV1) recovered energy metabolism and increased immune functions [9]. Metabolic boosting with TRPV1 agonist also decreased Aβ pathology and reversed memory deficits in TRPV1-knockout APP/PS1 mice. However, the above pharmacological or global transgenic knockout approaches are not microglial cell-specific and could not directly determine changes of microglial energy metabolism and activity.

## Conclusion

The metabolic homeostasis between OxPHOS and glycolysis is important for the transition between restorative functions and damaging ones for many immune cells. Metabolic reprogramming is essential for microglia to regulate their effector responses to brain injury. However, during brain disease states, the homeostatic balance between OxPHOS and glycolysis is disrupted. Targeting the dysregulation of microglial immunometabolism emerges as a new therapeutic strategy for treating a variety of neurodegenerative diseases.

## Data Availability

All articles reviewed in this manuscript have been cited in the references.
